# *Callicarpa* Species from Central Vietnam: Essential Oil Compositions and Mosquito Larvicidal Activities

**DOI:** 10.3390/plants9010113

**Published:** 2020-01-16

**Authors:** Nguyen Huy Hung, Le Thi Huong, Nguyen Thanh Chung, Nguyen Thi Hoai Thuong, Prabodh Satyal, Nguyen Anh Dung, Thieu Anh Tai, William N. Setzer

**Affiliations:** 1 Center for Advanced Chemistry, Institute of Research and Development, Duy Tan University, 03 Quang Trung, Da Nang 50000, Vietnam; 2 School of Natural Science Education, Vinh University, 182 Le Duan, Vinh City 43000, Nghe An Province, Vietnam; lehuong223@gmail.com (L.T.H.); hoaithuongnguyen468@gmail.com (N.T.H.T.); nadungch8@gmail.com (N.A.D.); 3 Graduate University of Science and Technology, Vietnam Academy of Science and Technology, 18-Hoang Quoc Viet, Cau Giay, Hanoi 10072, Vietnam; chungpuhoat@gmail.com; 4 Aromatic Plant Research Center, 230 N 1200 E, Suite 102, Lehi, UT 84043, USA; psatyal@aromaticplant.org; 5 Department of Pharmacy, Duy Tan University, 03 Quang Trung, Da Nang 50000, Vietnam; anhtai0808qn@gmail.com; 6 Department of Chemistry, University of Alabama in Huntsville, Huntsville, AL 35899, USA

**Keywords:** Lamiaceae, *Callicarpa candicans*, *Callicarpa rubella*, *Aedes aegypti*, atractylone, β-bisabolene, germacrone

## Abstract

There are around 140 species in the genus *Callicarpa*, with 23 species occurring in Vietnam. The Vietnamese *Callicarpa* species have been poorly studied. In this work, the leaf essential oils of *C. bodinieri*, *C. candicans*, *C. formosana*, *C. longifolia*, *C. nudiflora*, *C. petelotii*, *C. rubella*, and *C. sinuata*, have been obtained from plants growing in central Vietnam. The chemical compositions of the essential oils were determined using gas chromatography – mass spectrometry. Mosquito larvicidal activities of the essential oils were carried out against *Aedes aegypti*. All of the *Callicarpa* leaf essential oils showed larvicidal activity, but two samples of *C. candicans* were particularly active with 48-h LC_50_ values of 2.1 and 3.8 μg/mL. *Callicarpa* *candicans* essential oil should be considered as a potential alternative mosquito control agent.

## 1. Introduction

There are around 140 species of *Callicarpa* L. distributed in tropical and subtropical locations [1]. The genus has been placed in either the Verbenaceae or the Lamiaceae, but is currently placed in Lamiaceae [2,3]. Members of the genus have been used as fish poisons and in herbal medicine [1,2]. In this work, we present the essential oil compositions of several *Callicarpa* species growing wild in central Vietnam. In addition, some of the essential oils were screened for mosquito larvicidal activity.

*Callicarpa bodinieri* H. Lév. is native to western and central China [3], Vietnam, Laos, Cambodia, and Thailand [4]. The plant is used in traditional Chinese medicine to treat hematemesis (oral decoction of the leaves) and to treat wounds and bruises (fresh leaves externally) [5]. Flavonoids, sterols, triterpenoids [2], and diterpenoids [6] have been characterized in the leaves of *C. bodinieri*.

*Callicarpa candicans* (Burm. f.) Hochr. is native to southeast Asia, including China (Quangdong, Hainan), Burma, Cambodia, India, Laos, the Philippines, Thailand, and Vietnam [3]. The plant has been used as a fish poison in the Philippines [7], India [8], and Thailand [9]. In Vietnamese traditional medicine, the plant is used to prepare a tonic, to treat diseases of the liver and stomach, and externally to treat skin problems, pimples and ulcerations [10]. In Thailand, the stem bark of *C. candicans* is used to treat skin inflammation and swelling [11], while in the Philippines, the plant is taken to treat abdominal troubles [7], and sore throat and tonsillitis in the Mariana Islands [12]. In Vietnam, *C. candicans* is used as a tonic for postpartum care in women, to treat liver and abdominal pain, and as a diuretic [13]. Diterpenoids, triterpenoids, and flavonoids have been isolated from *C. candicans* [9,10].

*Callicarpa formosana* Rolfe is found in southeastern China (including Taiwan) [14], Japan, the Philippines [3], and Vietnam [15,16]. In China, *C. formosana* is used to treat scrofula (mycobacterial cervical lymphadenitis), and goiter [5], to stop bleeding [17], and to treat pyogenic infections [18]. Several sesquiterpenoids, diterpenoids, triterpenoids, iridoids, and flavonoids have been isolated and characterized from *C. formosana* [14].

*Callicarpa longifolia* Lam. Ranges from southern China through Malesia to Australia and from India through southeast Asia, including Vietnam [3,4,15,16,19,20,21]. Leaves of *C. longifolia* are used in China to treat wounds [2], while in Vietnam the plant is used to treat fever, diarrhea, abdominal pain, and as a tonic for postpartum women [13]. Kaurane diterpenoids and several flavonoids have been isolated from the leaves of *C. longifolia* [2,5].

*Callicarpa nudiflora* Hook. & Arn. is distributed from southern China through Southeast Asia as well as Burma, India, and Sri Lanka [3]. In Chinese traditional medicine, *C. nudiflora* is used for gastrointestinal bleeding, tuberculosis, upper respiratory tract infection, pneumonia, and bronchitis [5]. In Vietnam, the plant has been used traditionally for treating stomach bleeding and hepatitis [13]. The phytochemistry of *C. nudiflora* has been extensively studied. Terpenoids, including iridoids, diterpenoids, triterpenoids, as well as numerous flavonoids and phenylpropanoids have been isolated and identified in the plant [5].

*Callicarpa petelotii* Dop is endemic to Vietnam and recorded in the provinces of Lạng Sơn, Vĩnh Phúc, Hòa Bình, and Nghệ An [15,16]. There are no reports in the literature regarding ethnobotanical uses of the plant nor are there any phytochemical analyses reported.

*Callicarpa rubella* Lindl. ranges from southeastern China south through Burma, Thailand, Laos, and Vietnam [3]. In Vietnam, the fresh leaves are applied externally to treat scabies [13] or chewed to treat gum disease [22].

*Callicarpa sinuata* A.L. Budantzev & Phuong is endemic to Vietnam. It has been recorded in Quảng Bình province, (Vĩnh Linh: Do Linh), Sơn Trà peninsula (Đà Nãng City), and Gia Lai province [15,16]. There are no reports in the literature on the ethnobotany or phytochemistry of this species.

Mosquito-borne diseases have been a chronic menace to humans throughout history. *Aedes aegypti* (L.) (Diptera: Culicidae) is an important insect vector of arboviruses such as dengue [23], yellow fever [24], chikungunya [25], and Zika [26]. Dengue fever is widespread in Vietnam and epidemics are becoming more frequent [27]. Furthermore, chikungunya and Zika infections have recently been reported in Vietnam [28]. *Culex quinquefasciatus* Say (Diptera: Culicidae) is a vector of lymphatic filariasis [29] as well as several arboviruses such as West Nile virus and St. Louis encephalitis virus [30] and possibly Zika virus [31].

Insecticide resistance in *Aedes* and *Culex* mosquitoes has been growing throughout the world and may lead to an increase in the frequency of mosquito-borne diseases [32,33,34,35,36]. In addition to insecticide resistance, there is a chronic problem of the environmental impacts of synthetic insecticides [37,38], and there is a need for new and complementary methods for controlling insect vectors. Essential oils have shown promise as renewable and environmentally-safe alternatives to the use of synthetic insecticides [39,40,41,42,43]. As part of our continuing research on essential oils of aromatic plants from Vietnam and our search for natural mosquito control agents, we have collected and analyzed the essential oils from several species of *Callicarpa* growing wild in central Vietnam, and, depending on availability, the essential oils were screened for larvicidal activity against *Ae. aegypti*, and/or *Cx. quinquefasciatus*. The volatile components of *C. candicans*, *C. longifolia*, *C. petelotii*, and *C. sinuata* are reported for the first time. As far as we are aware, none of the *Callicarpa* essential oils presented in this work has been previously investigated in terms of mosquito larvicidal activity.

## 2. Results and Discussion

### 2.1. Essential Oil Compositions

#### 2.1.1. *Callicarpa bodinieri*

The leaf essential oil of *C. bodinieri* was obtained from Ngoc Linh Nature Reserve, Quang Nam province. The essential oil composition is presented in Table 1. The major components in *C. bodinieri* leaf essential oil were caryophyllene oxide (9.8%), β-selinene (8.9%), limonene (8.0%), and α-copaene (5.4%). A total of 106 compounds were identified in the essential oil accounting for 96.2% of the composition with sesquiterpene hydrocarbons (34.2%) and oxygenated sesquiterpenoids (37.8%) making up the bulk of the composition. The volatiles, obtained by head-space solid-phase micro extraction, of *C. bodinieri* from China have been reported [44]. The main volatile compounds were eremophila-1(10),11-diene (30.1%), cadina-3,9-diene (15. 2%), and longifolene (5.7%), and therefore, very different from the composition of the leaf essential oil from Vietnam.

#### 2.1.2. *Callicarpa candicans*

The leaf essential oils of *C. candicans* have been obtained from three different locations in Central Vietnam, Nghia Dan district (Nghe An province), Dai Loc district (Quang Nam province), and Hoa Vang district (Da Nang city). The *C. candicans* leaf essential oils were dominated by sesquiterpene hydrocarbons and oxygenated sesquiterpenes. (*E*)-Caryophyllene (19.0%, 7.1%, and 15.3%), β-selinene (6.2%, 5.7%, and 4.5%), caryophyllene oxide (2.9%, 13.4%, and 3.4%), and atractylone (37.7%, 4.2%, and 42.4%), respectively, for the samples from Nghia Dan, Dai Loc, and Hoa Vang, were the major components (Table 2). The stem bark essential oil, collected from Hoa Vang, was also rich in (*E*)-caryophyllene (7.8%), β-selinene (7.9%), caryophyllene oxide (11.1%), and atractylone (6.2%) (Table 3). As far as we are aware, there have been no previous reports on *C. candicans* essential oils.

#### 2.1.3. *Callicarpa formosana*

The leaf essential oil of *C. formosana* from Vietnam was dominated by caryophyllene oxide (38.9%), β-bisabolene (18.6%), and (*E*)-caryophyllene (6.5%) (Table 4). The composition of the essential oil from Vietnam is notably different from that collected in Guangdong, China, which was composed largely of spathulenol (20.2%), (*E*)-caryophyllene (17.2%), germacrene D (8.1%), and β-eudesmol (5.5%) [45].

#### 2.1.4. *Callicarpa longifolia*

Leaf essential oils of *C. longifolia* were obtained from Son Tra Peninsula (Da Nang City) and from Nghia Dan district (Nghe An province). Sesquiterpene hydrocarbons and oxygenated sesquiterpenoids dominated both essential oils (Table 5). There were, however, notable differences in the chemical profiles. For example, β-selinene was relatively abundant in the Nghia Dan sample (13.2%), but much less in the sample from Da Nang (3.2%). Conversely, *trans*-β-guaiene was abundant in the Da Nang sample (22.2%), but much lower in the Nghia Dan sample (0.4%). To our knowledge, there are no previous reports on the essential oil of *C. longifolia*.

#### 2.1.5. *Callicarpa nudiflora*

Unlike the essential oils of other *Callicarpa* species in this investigation, the leaf essential oil of *C. nudiflora* was dominated by the monoterpenes α-pinene (8.1%) and β-pinene (34.2%). Caryophyllene oxide (20.1%) was also an abundant component (Table 6). The chemical composition of Vietnamese *C. nudiflora* is markedly different from the leaf essential oil from China [46]. The Chinese sample showed only small quantities of α- and β-pinene (0.1% and 1.6%, respectively) and caryophyllene oxide was not observed. Conversely, humulene epoxide II was abundant in the sample from China (17.3%), but relatively minor in the sample from Vietnam (0.5%). Bisabolene oxide was abundant in the Chinese essential oil (10.5%), but was not detected in the sample from Vietnam.

#### 2.1.6. *Callicarpa petelotii*

Leaves of *C. petelotii* were collected from Tay Giang district, Quang Nam province, Vietnam. The leaf essential oil was dominated by the sesquiterpene hydrocarbons α-humulene (53.8%) and α-selinene (12.8%), in addition to humulene epoxide II (8.1%) (Table 7). There are no previous reports on the essential oil of *C. petelotii*.

#### 2.1.7. *Callicarpa rubella*

The leaf essential oils of *C. rubella* were obtained from three different sites in central Vietnam, Nậm Giải Commune (Quế Phong district, Pu Hoat Nature Reserve, Nghe An province), Bach Ma National Park (Phu Loc district, Thua Thien Hue province), and Tay Giang district (Quang Nam province). The essential oil compositions showed very different profiles (Table 8). The leaf essential oil from Nam Giai was dominated by caryophyllene oxide (25.1%), *cis*-thujopsenol (8.8%), and corymbolone (5.6%); β-bisabolene (25.0%), germacrone (22.1%), and (*E*)-caryophyllene (7.1%) were the major components of the leaf essential oil from Bach Ma; and the essential oil from Tay Giang was rich in (*E*)-caryophyllene (18.0%) and α-cubebene (17.4%). The volatiles, obtained by head-space solid-phase microextraction (HS-SPME) techniques, of *C. rubella* from China showed α-cubebene (8.7%), palmitic acid (5.4%), epizonarene (4.8%), heptadecane (4.8%), and spathulenol (4.5%) as the major components [47]. Thus, there is wide variation in the chemical compositions of *C. rubella* leaf essential oils. In addition to geographical and climatic effects, genetic differences may be responsible for the wide variation in essential oil composition; the Missouri Botanical Garden lists 11 subordinate taxa for *C. rubella* [3]. The stem bark essential oil from Bach Ma National Park was similar in composition to the leaf essential oil from that collection site. The major components in the bark essential oil were germacrone (23.9%), β-bisabolene (17.9%), germacrene B (8.4%), and (*E*)-caryophyllene (7.3%) (Table 9).

#### 2.1.8. *Callicarpa sinuata*

The leaf essential oil of *C. sinuata* from Son Tra Peninsula (Da Nang City) showed α-humulene (24.8%), α-copaene (12.6%), humulene epoxide II (6.7%), and spathulenol (5.9%) as the major components (Table 10). There have been no previous reports on the essential oil composition of *C. sinuata*.

With the exception of *C. nudiflora*, the *Callicarpa* leaf essential oils are dominated by sesquiterpene hydrocarbons and oxygenated sesquiterpenoids. Overall, the most abundant sesquiterpenes were (*E*)-caryophyllene and caryophyllene oxide and those compounds were found in all of the *Callicarpa* leaf essential oil samples. α-Humulene and β-selinene were also found in all of the leaf oil samples, while α-copaene, spathulenol, and humulene epoxide II were detected in 12 of the 13 leaf essential oils sampled. The furanoid sesquiterpenoid atractylone was only found in *C. candicans*.

### 2.2. Mosquito Larvicidal Activity

The 24-h and 48-h mosquito larvicidal activities of the *Callicarpa* leaf essential oil are summarized in Table 11 and Table 12. As far as we are aware, there have been no previous larvicidal investigations on these *Callicarpa* essential oils. Due to limited supply of some of the essential oils and limited supplies of mosquito larvae, not all essential oils could be screened against both mosquito species. Dias and Moraes have concluded that plant essential oils are considered larvicidal against *Ae. aegypti* if the LC_50_ values are less than 100 μg/mL [48]. Based on these guidelines, all of the *Callicarpa* essential oils showed good larvicidal activity. However, the leaf essential oils of *C. candicans*, from Nghia Dan district, Nghe An province and from Dai Loc district, Quang Nam province were particularly active with 48-h LC_50_ values of 3.8 and 2.1 μg/mL, respectively, against *Ae. aegypti*. The leaf essential oils of *C. candicans* were also effective larvicidal agents against *Cx. quinquefasciatus*.

The leaf essential oils of *C. candicans* were rich in (*E*)-caryophyllene, caryophyllene oxide, β-selinene and atractylone. Both (*E*)-caryophyllene and caryophyllene oxide have shown only weak larvicidal activity against *Ae. aegypti* [48]. However, atractylone may be contributing to the larvicidal activity; the compound has shown insecticidal [49] as well as acaricidal activity [50]. β-Selinene has also shown insecticidal activity [49]. In addition to the insecticidal properties of atractylone and β-selinene, there may be synergistic effects between these components and (*E*)-caryophyllene, caryophyllene oxide, or other minor components. Scalerandi and co-workers have shown that *Musca domestica* preferentially oxidize major essential oil components in a mixture while the components in lesser concentrations act as toxicants [51]. In addition, there were several unidentified components, particularly in the Dai Loc sample, that may be contributing to the larvicidal effects.

Interestingly, *C. nudiflora* leaf essential oil was rich in α- and β-pinene and caryophyllene oxide but was relatively inactive (24-h LC_50_ = 109 μg/mL) compared to the *C. candicans* leaf essential oils (24-h LC_50_ = 2.0 and 1.2 μg/mL) against *Cx. quinquefasciatus*. Consistent with these results, both α-pinene and β-pinene have shown relatively weak larvicidal activity against *Cx. quinquefasciatus* [52]. Likewise, the seed essential oil of *Psoralea corylifolia*, rich in caryophyllene oxide (40.8%), also showed relatively weak larvicidal activity against *Cx. quinquefasciatus* [53]. *C. nudiflora* leaf essential oil was also less active against *Ae. aegypti* larvae with a 24-h LC_50_ value of 37.5 μg/mL. There are conflicting results regarding the larvicidal activities of α- and β-pinene on *Ae. aegypti*. Lucia and co-workers reported LC_50_ values of 15.4 and 12.1 μg/mL for α- and β-pinene, respectively, against *Ae. aegypti* [54], while Waliwitiya and co-workers found the pinenes to be inactive (LC_50_ > 500 μg/mL) against the mosquito larvae [55]. Caryophyllene oxide is apparently only weakly larvicidal (LC_50_ = 125 μg/mL) on *Ae. aegypti* [39,56].

The leaf essential oil of *C. longifolia* from Nghia Dan, rich in (*E*)-caryophyllene (28.0%) and β-selinene (13.2%), showed larvicidal activity with 24-h LC_50_ of 37.4 μg/mL. (*E*)-Caryophyllene is relatively inactive with reported LC_50_ values of 93.7 [57] and 1202 μg/mL [56]. Notably, *Piper humaytanum* leaf essential oil, with 3.5% (*E*)-caryophyllene and 15.8% β-selinene, was weakly larvicidal (LC_50_ = 156 μg/mL) against *Ae. aegypti* [58].

The larvicidal activity of *C. bodinieri* leaf essential oil was the weakest of the *Callicarpa* species tested with a 24-h LC_50_ of 54 μg/mL. Limonene was one of the major components (8.0%), and this compound had shown larvicidal activity against *Ae. aegypti* of around 30 μg/mL [59]. Caryophyllene oxide, another major component (9.8%) is inactive against *Ae. aegypti* [39]. Although apparently not tested against mosquito larvae, β-selinene (8.9% in *C. bodinieri* leaf essential oil) is known to be insecticidal against *Drosophila melanogaster* adults [49].

The leaf essential oils of *C. formosana*, *C. rubella* (Nam Giai), *C. rubella* (Tay Giang), and *C. sinuata* showed comparable larvicidal activities with 24-h LC_50_ ranging from 24.2 to 31.9 μg/mL. However, the chemical compositions of the essential oils were very different.

**Figure 1:**
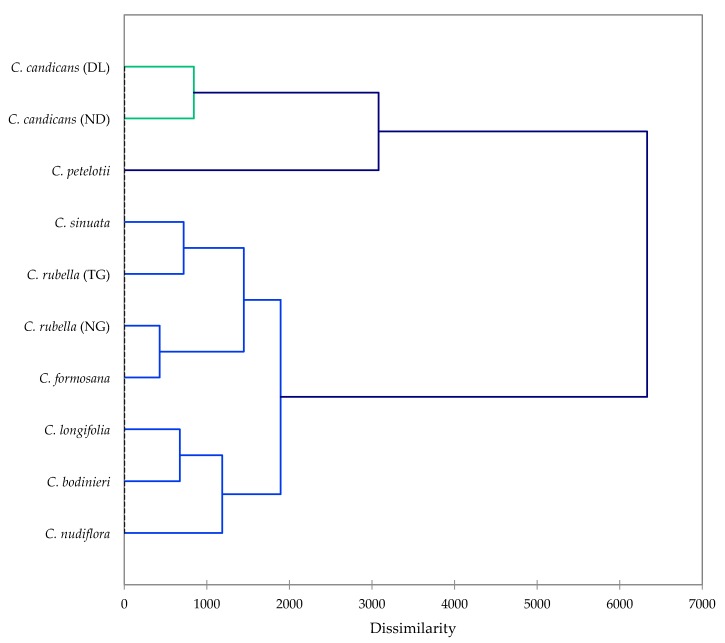
Agglomerative hierarchical cluster analysis based on the major components of the *Callicarpa* essential oils from central Vietnam along with larvicidal activities (LC_50_ and LC_90_) against *Aedes aegypti*.

In order to evaluate potential correlation between constituents and larvicidal activities, multivariate analyses (hierarchical cluster analysis, HCA, and principal component analysis, PCA, were undertaken. The hierarchical cluster analysis (Figure 1) showed four groupings. Group 1 is made up of the two *C. candicans* samples and represents a very larvicidal group (average 24-h LC_50_ and LC_90_ = 4.02 and 9.34 μg/mL). The major components in this group are atractylone (average 20.9%) and caryophyllene oxide (average 8.1%). Group 2 is a single sample, *C. petelotii* is somewhat active with 24-h LC_50_ and LC_90_ of 19.1 and 37.9 μg/mL and α-humulene, α-selinene, and humulene epoxide II as the major components. Group 3 (*C. sinuata*, *C. formosana*, and both *C. rubella* samples) had average 24-h larvicidal LC_50_ and LC_90_ of 27.7 and 50.9 μg/mL, respectively. The major component in group 3 is caryophyllene oxide with an average concentration of 17.1%. Group 4 is the least active group (24-h LC_50_ and LC_90_ = 43.0 and 73.0 μg/mL) and also has caryophyllene oxide as the major component (average = 12.0%) as well as (*E*)-caryophyllene (average = 10.6%).

**Figure 2:**
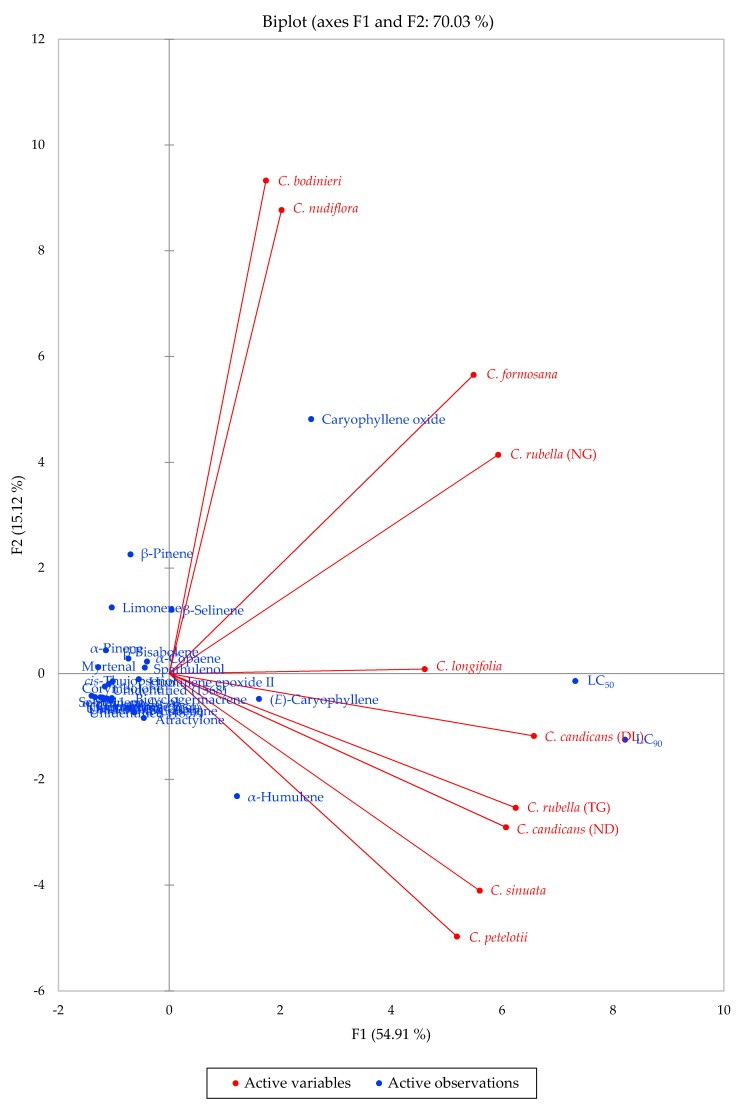
Principal component biplot of PC1 and PC2 scores and loadings indicating the correlation of chemical components of *Callicarpa* essential oils from central Vietnam and *Aedes aegypti* larvicidal activity.

The principal component analysis (Figure 2) does not reveal any clear associations between chemical components and larvicidal activity. (*E*)-Caryophyllene, caryophyllene oxide, and α-humulene were found in all of the samples and therefore correlate with the essential oil samples and not necessarily with the larvicidal activities. Apparently the synergistic and antagonistic interactions of the components in these essential oils are too subtle to be parsed out with so few data.

## 3. Materials and Methods 

### 3.1. Plant Material

Plant material (leaves and/or stem bark) from *Callicarpa* species was collected from several locations in central Vietnam (Table 13). The plant material from several individuals from each site were combined in order to provide enough plant material for each species. The plants were identified by Dr. Do Ngoc Dai, and voucher specimens (Table 13) have been deposited in the School of Natural Science Education, Vinh University. The fresh plant materials (2.0 kg each) were shredded and hydrodistilled for 4 h using a Clevenger type apparatus (Witeg Labortechnik, Wertheim, Germany). The yields of essential oils are summarized in Table 13.

### 3.2. Gas Chromatography-Mass Spectrometry

Each of the *Callicarpa* essential oils was analyzed by GC-MS using a Shimadzu GCMS-QP2010 Ultra (Shimadzu Scientific Instruments, Columbia, MD, USA) operated in the electron impact (EI) mode (electron energy = 70 eV), scan range = 40–400 atomic mass units, scan rate = 3.0 scans/s, and GC-MS solution software. The GC column was a ZB-5 fused silica capillary column (Phenomenex, Torrance, CA, USA) (30 m length × 0.25 mm internal diameter) with a (5% phenyl)-polymethylsiloxane stationary phase and a film thickness of 0.25 μm. The carrier gas was helium with a column head pressure of 552 kPa and flow rate of 1.37 mL/min. Injector temperature was 250 °C and the ion source temperature was 200 °C. The GC oven temperature program was programmed for 50 °C initial temperature, temperature increased at a rate of 2 °C/min to 260 °C. A 5% *w*/*v* solution of the sample in CH_2_Cl_2_ was prepared and 0.1 μL was injected with a splitting mode (30:1). Identification of the oil components was based on their retention indices determined by reference to a homologous series of *n*-alkanes (C_8_-C_40_), and by comparison of their mass spectral fragmentation patterns with those reported in the databases [60,61,62,63]. The percentages of each component in the essential oils are reported as raw percentages based on total ion current without standardization.

### 3.3. Mosquito Larvicidal Assay

Eggs of *Ae. aegypti* were purchased from Institute of Biotechnology, Vietnam Academy of Science and Technology, and maintained at the Laboratory of Department of Pharmacy of Duy Tan University, Da Nang, Vietnam. For the assay, aliquots of the essential oils of *Callicarpa* species, dissolved in DMSO (1% stock solution), was placed in a 500-mL beaker and added to water that contained 20 larvae (third and early fourth instar). With each experiment, a set of controls using DMSO was also run for comparison. Mortality was recorded after 24 h and again after 48 h of exposure during which no nutritional supplement was added. The experiments were carried out 25 ± 2 °C. Each test was conducted with four replicates with several concentrations (100, 50, 25, 12.5, 6.0, 3.0, 1.5, and 0.75 μg/mL). Larvicidal activity against *Culex quinquefasciatus* (The larvae were fed on Koi fish food: Adults were provided with a 10% sucrose solution and a 1-week-old chick for blood feeding.) were determined similarly with concentrations of 150, 100, 50, 25, 6.0, 3.0, 1.5, and 0.75 μg/mL. Permethrin was used as a positive control. The acute larvicidal effects on *Ae. aegypti*, and *Cx. quinquefasciatus* were recorded 24 h and 48 h after treatment. The data obtained were subjected to log-probit analysis [64] to obtain LC_50_ values, LC_90_ values, and 95% confidence limits using XLSTAT v. 2018.5 (Addinsoft, Paris, France).

### 3.4. Statistical Analysis

Mosquito larvicidal activities (LC_50_ and LC_90_) against *Ae. aegypti* and *Cx. quinquefasciatus* were determined by log-probit analysis using XLSTAT v. 2018.5 (Addinsoft, Paris, France). The more abundant chemical components of the *Callicarpa* essential oils were used in the multivariate analyses. The essential oil compositions were treated as operational taxonomic units (OTUs), and the concentrations (percentages) of 26 major essential oil components and the 24-h LC_50_ and LC_90_ larvicidal activity data were used to determine the associations between the *Callicarpa* essential oils using agglomerative hierarchical cluster (AHC) analysis using XLSTAT Premium, version 2018.5 (Addinsoft, Paris, France). Dissimilarity was determined using Euclidean distance, and clustering was defined using Ward’s method. For the principal component analysis (PCA), the 26 major components and the larvicidal data were taken as variables using a Pearson correlation matrix using XLSTAT Premium, version 2018.5 (Addinsoft, Paris, France). A total of 280 data (28 variables × 10 samples) were used for the PCA.

## 4. Conclusions

There are profound chemical variations in the leaf essential oils of *Callicarpa* species, both between species and within species. All of the *Callicarpa* leaf essential oils showed larvicidal activity against *Ae. aegypti*. However, *C. candicans* showed excellent mosquito larvicidal activity against *Ae. aegypti* as well as *Cx. quinquefasciatus*, which can be attributed to atractylone and/or to unidentified components. This essential oil, therefore, may represent a low-cost and environmentally friendly mosquito control agent. Nevertheless, although the larvicidal activities of *Callicarpa* leaf essential oils are promising, additional screening on non-target organisms is needed [41,42].

## Figures and Tables

**Table 1:** Chemical composition of *Callicarpa bodinieri* leaf essential oil from Ngoc Linh Nature Reserve, Vietnam.

RI ^a^	RI ^b^	Compound	%
923	924	α-Thujene	tr ^c^
930	932	α-Pinene	1.5
945	945	α-Fenchene	tr
947	946	Camphene	0.1
970	969	Sabinene	tr
975	974	β-Pinene	1.7
977	974	1-Octen-3-ol	tr
983	979	Octan-3-one	tr
984	984	*p*-Menth-3-ene	tr
986	988	Myrcene	0.5
1002	998	Octanal	0.3
1022	1024	*p*-Cymene	0.2
1027	1024	Limonene	8.0
1030	1026	1,8-Cineole	0.1
1068	1063	1-Octanol	0.2
1089	1082	*m*-Cymenene	0.1
1098	1095	Linalool	0.3
1103	1100	Nonanal	2.3
1120	1119	*trans*-*p*-Mentha-2,8-dien-1-ol	0.1
1125	1122	α-Campholenal	0.1
1131	1132	*cis*-Limonene oxide	0.2
1135	1133	*cis*-*p*-Mentha-2,8-dien-1-ol	0.1
1135	1137	*trans*-Limonene oxide	0.1
1137	1135	Nopinone	tr
1139	1135	*trans*-Pinocarveol	0.1
1183	1178	Naphthalene	0.5
1186	1187	*trans*-*p*-Mentha-1(7),8-dien-2-ol	0.2
1194	1195	Myrtenal	0.3
1204	1201	Decanal	0.3
1217	1215	*trans*-Carveol	0.2
1242	1239	Carvone	0.2
1260	1260	Dec-(2*E*)-enal	0.1
1265	1267	Nonanoic acid	0.4
1281	1287	Bornyl acetate	0.1
1286	1287	Dihydroedulan IA	0.3
1291	1294	Dihydroedulan IIA	0.3
1295	1298	(*Z*)-Theaspirane	0.2
1300	1300	Tridecane	0.1
1305	1305	Undecanal	0.1
1308	1310	(*Z*)-Patchenol	tr
1312	1314	(*E*)-Theaspirane	0.1
1344	1345	α-Cubebene	0.1
1349	1671	1-Tetradecanol	0.1
1366	1373	α-Ylangene	0.4
1373	1374	α-Copaene	5.4
1381	1387	β-Bourbonene	0.1
1386	1389	β-Elemene	0.9
1415	1419	β-Ylangene	0.4
1416	1417	β-Caryophyllene	1.0
1424	1430	γ-Maaliene	0.3
1427	1431	β-Gurjunene (= Calarene)	2.3
1436	1439	Aromadendrene	2.3
1442	1447	Selina-5,11-diene	0.2
1449	1455	Valerena-4,7(11)-diene	0.8
1452	1452	α-Humulene	0.6
1457	1458	*allo*-Aromadendrene	1.8
1470	1475	Selina-4,11-diene	0.2
1472	1478	γ-Muurolene	2.7
1476	1483	α-Amorphene	0.2
1478	1479	*ar*-Curcumene	0.1
1486	1489	β-Selinene	8.9
1489	1495	γ-Amorphene	0.1
1493	1498	α-Selinene	0.9
1495	1500	α-Muurolene	0.6
1504	1505	β-Bisabolene	0.2
1510	1513	γ-Cadinene	1.0
1512	1514	Cubebol	0.1
1515	1522	δ-Cadinene	0.2
1517	1521	*trans*-Calamenene	0.5
1518	1528	*cis*-Calamenene	0.7
1538	1544	α-Calacorene	0.6
1558	1561	(*E*)-Nerolidol	0.6
1559	1564	β-Calacorene	0.8
1567	1566	Maaliol	0.5
1567	1567	Palustrol	0.6
1574	1577	Spathulenol	2.3
1579	1582	Caryophyllene oxide	9.8
1583	1590	Globulol	3.8
1585	1590	β-Copaen-4α-ol	1.3
1590	1594	Salvial-4(14)-en-1-one	0.6
1591	1592	Viridiflorol	2.2
1594	1595	Cubeban-11-ol	0.7
1599	1598	Dehydroxy-*iso*-calamendiol	0.4
1601	1602	Ledol	0.7
1604	1600	Rosifoliol	0.5
1607	1608	Humulene epoxide II	2.6
1612	1618	1,10-di-*epi*-Cubenol	0.5
1623	1630	Muurola-4,10(14)-dien-1β-ol	1.7
1625	1627	1-*epi*-Cubenol	0.6
1631	1642	Caryophylla-4(12),8(13)-dien-5α-ol	0.5
1634	1644	Caryophylla-4(12),8(13)-dien-5β-ol	0.4
1639	1638	τ-Cadinol	0.5
1641	1640	τ-Muurolol	0.6
1644	1644	α-Muurolol (= δ-Cadinol)	0.5
1653	1652	α-Cadinol	0.9
1655	1651	Pogostol	2.5
1660	1668	*ar*-Turmerone	0.5
1662	1668	*trans*-Calamenen-10-ol	0.3
1668	1668	14-Hydroxy-9-*epi*-(*E*)-caryophyllene	0.4
1670	1675	Cadalene	0.6
1682	1685	Germacra-4(15),5,10(14)-trien-1α-ol	0.3
1806	1816	Callicarpenal	0.4
1837	1841	Phytone	1.5
1955	1958	Palmitic acid	0.7
2103	2109	(*E*)-Phytol	1.8
2700	2700	Heptacosane	0.6
		Monoterpene hydrocarbons	12.1
		Oxygenated monoterpenoids	2.1
		Sesquiterpene hydrocarbons	34.2
		Oxygenated sesquiterpenoids	37.8
		Diterpenoids	3.3
		Others	6.6
		Total identified	96.2

^a^ RI = Retention Index determined with respect to a homologous series of n-alkanes on a ZB-5 column. ^b^ Retention indices from the databases. ^c^ tr = trace (<0.05%).

**Table 2:** Chemical compositions of *Callicarpa candicans* leaf essential oils from Vietnam.

RI ^a^	RI ^b^	Compound	%		
			Nghia Dan	Dai Loc	Hoa Vang
873	863	2,3-Dimethyl-cyclohexa-1,3-diene	---	tr ^c^	---
930	932	α-Pinene	---	tr	---
975	974	β-Pinene	---	0.1	---
977	974	1-Octen-3-ol	0.3	0.1	0.1
983	979	3-Octanone	0.2	tr	0.1
996	988	3-Octanol	---	tr	tr
1024	1024	*p*-Cymene	---	0.4	tr
1027	1024	Limonene	---	0.1	---
1030	1026	1,8-Cineole	---	tr	---
1068	1067	*cis*-Linalool oxide (furanoid)	---	tr	---
1083	1086	Terpinolene	---	tr	---
1084	1084	*trans*-Linalool oxide (furanoid)	---	tr	---
1089	1087	2-Nonanone	0.1	0.1	0.1
1099	1095	Linalool	0.6	1.4	0.4
1182	1178	Naphthalene	---	0.3	---
1191	1190	Methyl salicylate	---	0.1	0.1
1287	1287	Dihydroedulan IA	0.1	0.2	0.1
1291	1293	2-Undecanone	0.2	0.1	0.3
1295	1310	(*Z*)-Theaspirane	---	tr	---
1312	1314	(*E*)-Theaspirane	---	tr	---
1335	1335	δ-Elemene	0.2	1.1	0.1
1335	1330	(*Z*)-Jasmone	---	0.1	---
1344	1346	α-Terpinyl acetate	---	0.1	---
1366	1373	α-Ylangene	---	0.1	---
1376	1374	α-Copaene	0.1	0.1	---
1376	1383	(*E*)-β-Damascenone	---	tr	---
1381	1383	*cis*-β-Elemene	---	---	0.1
1388	1390	*trans*-β-Elemene	0.9	1.5	1.7
1401	1408	(*Z*)-Caryophyllene	---	0.1	---
1410	1411	Thymohydroquinone dimethyl ether	---	0.1	---
1419	1417	(*E*)-Caryophyllene	19.0	7.1	15.3
1428	1434	γ-Elemene	3.2	0.5	2.3
1437	1439	Aromadendrene	0.2	0.2	0.1
1451	1454	(*E*)-β-Farnesene	---	---	0.1
1455	1452	α-Humulene	2.4	1.2	1.9
1459	1458	*allo*-Aromadendrene	0.2	0.1	0.1
1470	1475	Selina-4,11-diene	---	0.2	---
1474	1476	β-Chamigrene	0.1	0.1	0.1
1475	1478	γ-Muurolene	0.3	0.1	---
1478	1483	α-Amorphene	0.3	0.3	0.2
1480	1487	(*E*)-β-Ionone	---	0.7	---
1481	1484	Germacrene D	0.9	---	0.5
1484	1487	Aristolochene	0.1	---	---
1488	1489	β-Selinene	6.2	5.7	4.5
1493	1499	Curzerene	2.2	0.8	5.3
1495	1498	α-Selinene	---	1.0	1.7
1496	1500	Bicyclogermacrene	3.0	---	---
1498	1500	α-Muurolene	0.2	---	---
1504	1505	(*E*,*E*)-α-Farnesene	0.7	---	0.4
1513	1513	γ-Cadinene	0.3	---	---
1518	1522	δ-Cadinene	0.4	---	0.1
1519	1520	7-*epi*-α-Selinene	0.1	---	0.1
1533	1528	Zonarene	0.1	---	---
1534	1540	Selina-4(15),7(11)-diene	1.5	1.8	0.9
1541	1545	Selina-3,7(11)-diene	0.5	0.9	0.2
1546	1548	α-Elemol	---	0.4	0.1
1556	1559	Germacrene B	6.1	0.1	5.1
1557	1561	(*E*)-Nerolidol	---	0.3	---
1576	1577	Spathulenol	0.7	2.1	1.0
1581	1582	Caryophyllene oxide	2.9	13.4	3.4
1583	1590	Globulol	---	0.1	---
1593	1594	Salvial-4(14)-en-1-one	0.1	---	---
1598	1601	*trans*-β-Elemenone	0.1	---	---
1609	1608	Humulene epoxide II	0.3	1.6	0.4
1627	1629	*iso*-Spathulenol	0.1	0.3	0.1
1630	1642	Caryophylla-4(12),8(13)-dien-5α-ol	---	0.4	---
1636	1644	Caryophylla-4(12),8(13)-dien-5β-ol	1.2	1.1	1.3
1643	1644	α-Muurolol (= Torreyol)	0.1	---	---
1647	1642	Selina-3,11-dien-6α-ol	---	---	tr
1655	1649	β-Eudesmol	1.9	3.6	1.9
1662	1657	Atractylone	37.7	4.2	42.4
1666	1666	Intermedeol	0.1	---	---
1668	1668	14-Hydroxy-9-*epi*-(*E*)-caryophyllene	0.2	2.5	0.4
1693	1693	Germacrone	0.3	---	0.2
1696	1700	Eudesm-7(11)-en-4-ol	---	---	tr
1711	---	Unidentified ^d^	0.2	1.1	0.4
1713	1713	Longifolol	---	---	0.1
1736	1734	1(10),11-Eremophiladien-9-one	0.5	---	---
1739	1746	8α,11-Elemodiol	---	---	0.1
1768	---	Unidentified ^e^	---	1.0	0.3
1799	1796	(*E*)-Isovalencenol	---	---	0.1
1858	---	Unidentified ^f^	0.6	10.9	1.8
1919	---	Unidentified ^g^	---	1.0	---
1936	---	Unidentified ^h^	---	1.0	---
1994	1994	Manoyl oxide	0.9	3.3	---
1998	1997	Kaur-15-ene	0.1	---	---
2005	---	Unidentified ^i^	0.5	3.6	0.9
2055	---	Unidentified ^j^	0.3	6.6	0.8
2091	---	Unidentified ^k^	---	8.5	1.0
2105	2109	(*E*)-Phytol	---	---	0.8
		Monoterpene hydrocarbons	0.0	0.6	0.0
		Oxygenated monoterpenoids	0.6	1.6	0.4
		Sesquiterpene hydrocarbons	49.0	22.6	40.7
		Oxygenated sesquiterpenoids	46.0	29.9	51.5
		Diterpenoids	1.0	3.3	0.8
		Others	0.8	1.5	0.8
		Total Identified	97.3	59.7	94.2

^a^ RI = Retention Index determined with respect to a homologous series of n-alkanes on a ZB-5 column. ^b^ Retention indices from the databases. ^c^ tr = trace (<0.05%). ^d^ MS: 220(41%), 205(20%), 202(30%), 187(30%), 162(92%), 158(33%), 149(63%), 147(61%), 121(79%), 119(84%), 107(79%), 105(73%), 97(49%), 93(61%), 91(71%), 79(48%), 77(38%), 67(53%), 55(49%), 43(100%), 41(62%). ^e^ MS: 220(64%), 202(9%), 177(100%), 159(77%), 135(49%), 123(74%), 107(83%), 93(64%), 81(58%), 67(53%), 55(55%), 43(55%), 41(65%). ^f^ MS: 233(16%), 232(100%), 204(19%), 189(21%), 161(29%), 148(31%), 147(38%), 135(32%), 134(33%), 133(44%), 121(34%), 108(48%), 105(37%), 93(53%), 91(51%), 79(44%), 77(33%), 67(22%), 55(24%), 53(23%), 41(34%). ^g^ MS: 236(12%), 222(4%), 203(4%), 193(25%), 175(25%), 161(9%), 149(22%), 147(48%), 133(14%), 121(13%), 119(13%), 107(19%), 105(26%), 93(24%), 91(27%), 79(22%), 67(17%), 55(15%), 43(100%), 41(22%). ^h^ MS: 290(23%), 165(6%), 151(100%), 138(12%), 123(25%), 109(24%), 95(17%), 81(26%), 69(35%), 55(23%), 43(14%), 41(20%). ^i^ MS: 230(70%), 215(100%), 201(44%), 187(30%), 174(33%), 160(31%), 159(34%), 145(27%), 131(27%), 117(20%), 115(18%), 105(27%), 91(50%), 79(28%), 77(32%), 53(31%), 41(27%). ^j^ MS: 233(17%), 232(100%), 217(29%), 204(15%), 189(12%), 187(12%), 176(13%), 161(19%), 148(16%), 147(23%), 133(26%), 122(51%), 121(45%), 107(52%), 105(42%), 93(63%), 91(59%), 79(57%), 77(39%), 67(29%), 55(27%), 53(36%), 41(45%). ^k^ MS: 248(4%), 230(8%), 220(16%), 205(16%), 191(15%), 175(18%), 159(14%), 147(100%), 133(16%), 121(42%), 119(33%), 107(28%), 105(40%), 93(35%), 91(45%), 79(41%), 67(24%), 55(21%), 53(24%), 41(35%).

**Table 3:** Chemical composition of *Callicarpa candicans* stem bark essential oil from Hoa Vang, Vietnam.

RI ^a^	RI ^b^	Compound	%
978	974	1-Octen-3-ol	1.4
996	988	3-Octanol	0.4
1099	1095	Linalool	0.6
1191	1190	Methyl salicylate	0.9
1335	1336	Bicycloelemene	1.6
1350	1356	Eugenol	0.4
1389	1389	β-Elemene	0.6
1419	1417	β-Caryophyllene	7.8
1429	1434	γ-Elemene	1.1
1455	1452	α-Humulene	1.4
1478	1483	α-Amorphene	0.2
1483	1476	β-Chamigrene	1.5
1487	1496	Indipone	0.8
1489	1489	β-Selinene	7.9
1493	1498	Curzerene	0.6
1496	1498	α-Selinene	2.0
1519	1520	7-*epi*-α-Selinene	0.6
1537	1528	Zonarene	2.6
1541	1545	Selina-3,7(11)-diene	1.1
1559	1559	Germacrene B	1.6
1560	1561	(*E*)-Nerolidol	0.5
1576	1577	Spathulenol	1.5
1582	1582	Caryophyllene oxide	11.1
1609	1608	Humulene epoxide II	1.3
1617	---	Unidentified ^c^	1.2
1636	1644	Caryophylla-4(12),8(13)-dien-5β-ol	1.2
1647	1642	Selina-3,11-dien-6α-ol	0.4
1654	1652	α-Eudesmol	5.3
1659	1657	Atractylone	6.2
1670	1668	14-Hydroxy-9-*epi*-(*E*)-caryophyllene	1.4
1692	1693	Germacrone	0.3
1711	---	Unidentified ^d^	1.2
1730	1728	*iso*-Longifolol	0.8
1735	1734	1(10),11-Eremophiladien-9-one	1.2
1770	---	Unidentified ^e^	1.1
1858	---	Unidentified ^f^	4.1
1985	---	Unidentified ^g^	2.2
1985	1987	1-Eicosene	2.2
1993	1994	Manoyl oxide	6.2
1997	1997	Kaur-15-ene	0.4
2006	---	Unidentified ^h^	2.9
2054	---	Unidentified ^i^	2.9
2089	---	Unidentified ^j^	5.1
2106	2109	(*E*)-Phytol	0.3
		Monoterpene hydrocarbons	0.0
		Oxygenated monoterpenoids	0.6
		Sesquiterpene hydrocarbons	30.7
		Oxygenated sesquiterpenoids	31.8
		Diterpenoids	6.9
		Others	3.1
		Total Identified	73.1

^a^ RI = Retention Index determined with respect to a homologous series of n-alkanes on a ZB-5 column. ^b^ Retention indices from the databases. ^c^ MS: 207(45%), 204(53%), 189(54%), 161(39%), 147(32%), 137(30%), 135(68%), 133(31%), 123(41%), 109(47%), 95(53%), 93(50%), 81(100%), 71(60%), 67(43%), 55(45%), 43(97%), 41(37%). ^d^ MS: 220(49%), 205(29%), 202(48%), 187(40%), 162(100%), 159(41%), 149(53%), 147(58%), 131(37%), 121(77%), 119(85%), 107(68%), 105(68%), 97(47%), 93(57%), 91(62%), 79(40%), 77(31%), 67(43%), 55(46%), 43(79%), 41(48%). ^e^ MS: 220(46%), 202(9%), 187(10%), 177(100%), 159(50%), 138(27%), 135(37%), 123(58%), 107(57%), 95(36%), 93(40%), 91(37%), 81(35%), 79(29%), 67(19%), 55(29%), 43(30%), 41(29%). ^f^ MS: 233(17%), 232(100%), 217(8%), 204(22%), 189(24%), 176(17%), 161(26%), 148(26%), 147(27%), 135(27%), 134(30%), 133(38%), 122(26%), 121(30%), 108(40%), 105(30%), 93(47%), 91(41%), 79(35%), 77(25%), 67(17%), 55(19%), 53(17%), 41(24%). ^g^ MS: 236(2%), 221(3%), 218(5%), 203(7%), 182(24%), 179(27%), 162(19%), 161(28%), 143(45%), 234(30%), 125(60%), 123(64%), 121(43%), 109(55%), 107(39%), 97(53%), 95(63%), 93(47%), 81(70%), 79(37%), 71(40%), 69(69%), 67(40%), 55(100%), 43(87%), 41(70%). ^h^ MS: 230(70%), 215(100%), 201(49%), 187(31%), 174(33%), 160(25%), 159(27%), 145(21%), 131(27%), 117(15%), 105(19%), 91(36%), 79(21%), 77(23%), 55(14%), 53(23%), 41(18%). ^i^ MS: 233(18%), 232(100%), 217(31%), 204(18%), 190(14%), 187(14%), 176(13%), 161(15%), 148(14%), 147(21%), 133(21%), 122(49%), 121(38%), 107(44%), 105(34%), 93(55%), 91(46%), 79(43%), 77(28%), 67(23%), 55(22%), 53(29%), 41(30%). ^j^ MS: 342(1%), 248(4%), 230(27%), 220(21%), 215(35%), 205(20%), 203(19%), 191(18%), 175(24%), 159(22%), 147(100%), 133(20%), 131(19%), 121(45%), 119(36%), 105(48%), 91(50%), 79(43%), 77(30%), 67(26%), 55(25%), 53(29%), 43(17%), 41(35%).

**Table 4:** Chemical composition of *Callicarpa formosana* leaf essential oil from Ngoc Linh Nature Reserve, Vietnam.

RI ^a^	RI ^b^	Compound	%
930	932	α-Pinene	0.6
975	974	β-Pinene	0.3
1022	1024	*p*-Cymene	0.1
1026	1024	Limonene	0.1
1097	1095	Linalool	0.1
1103	1100	Nonanal	0.1
1182	1187	(3*Z*)-Hexenyl butyrate	0.1
1188	1195	Hexyl butyrate	0.1
1192	1197	(2*E*)-Hexenyl butyrate	0.1
1227	1221	(3Z)-Hexenyl 2-methylbutyrate	0.1
1232	1236	Hexyl 2-methylbutyrate	tr ^c^
1234	1235	(2*E*)-Hexenyl 2-methylbutyrate	0.1
1285	1287	Dihydroedulan IA	0.2
1290	1294	Dihydroedulan IIA	0.1
1295	1298	(*Z*)-Theaspirane	0.3
1311	1314	(*E*)-Theaspirane	0.3
1372	1374	α-Copaene	0.1
1385	1389	β-Elemene	0.2
1398	1402	α-Funebrene	0.1
1400	1408	(*Z*)-Caryophyllene	0.1
1412	1410	α-Cedrene	tr
1416	1417	(*E*)-Caryophyllene	6.5
1426	1430	β-Copaene	tr
1430	1428	Dictamnol	tr
1443	1453	Geranyl acetone	0.1
1448	1454	(*E*)-β-Farnesene	0.2
1452	1452	α-Humulene	0.6
1471	1478	γ-Muurolene	0.1
1477	1479	*ar*-Curcumene	0.6
1484	1491	Eremophilene	0.1
1485	1489	β-Selinene	0.1
1491	1498	α-Selinene	0.2
1494	1500	α-Muurolene	0.1
1503	1505	β-Bisabolene	18.6
1509	1511	Sesquicineole	0.3
1517	1521	*trans*-Calamenene	0.2
1519	1521	β-Sesquiphellandrene	0.1
1539	1542	*cis*-Sesquisabinene hydrate	0.2
1547	---	Unidentified ^d^	2.1
1550	1555	*cis*-7-*epi*-Sesquisabinene hydrate	0.3
1556	1561	(*E*)-Nerolidol	0.5
1579	1582	Caryophyllene oxide	38.9
1603	---	Unidentified ^e^	1.8
1606	1608	Humulene epoxide II	1.5
1616	---	Unidentified ^f^	1.1
1624	1627	1-*epi*-Cubenol	0.2
1629	1642	Caryophylla-4(12),8(13)-dien-5α-ol	0.6
1633	1644	Caryophylla-4(12),8(13)-dien-5β-ol	1.1
1643	1644	α-Muurolol (= δ-Cadinol)	1.0
1652	1656	14-Hydroxy-9-*epi*-(*Z*)-caryophyllene	0.5
1654	1651	Pogostol	0.9
1659	1668	*ar*-Turmerone	0.3
1667	1668	14-Hydroxy-9-*epi*-(*E*)-caryophyllene	1.1
1676	1678	9-Tetradecyn-1-ol	0.4
1681	1683	*epi*-α-Bisabolol	0.8
1683	1685	α-Bisabolol	1.8
1722	---	Unidentified ^g^	1.4
1809	---	Unidentified ^h^	1.3
1830	1836	Neophytadiene	0.2
1835	1841	Phytone	0.8
1939	1947	*iso*-Phytol	0.1
1652	1958	Palmitic acid	0.2
2101	2109	(*E*)-Phytol	3.5
2131	---	Unidentified ^i^	1.7
		Monoterpene hydrocarbons	1.0
		Oxygenated monoterpenoids	0.1
		Sesquiterpene hydrocarbons	28.0
		Oxygenated sesquiterpenoids	50.5
		Diterpenoids	4.7
		Others	1.5
		Total Identified	85.9

^a^ RI = Retention Index determined with respect to a homologous series of n-alkanes on a ZB-5 column. ^b^ Retention indices from the databases. ^c^ tr = trace (<0.05%). ^d^ MS: 205(5%), 187(5%), 176(7%), 163(9%), 149(12%), 138(23%), 120(22%), 109(28%), 107(37%), 106(91%), 93(50%), 91(68%), 79(100%), 69(33%), 67(32%), 55(30%), 43(61%), 41(65%). ^e^ MS: 205(11%), 187(10%), 159(34%), 148(16%), 131(19%), 121(32%), 119(39%), 105(41%), 93(68%), 91(43%), 81(34%), 79(52%), 69(34%), 67(34%), 59(35%), 43(100%), 41(44%). ^f^ MS: 202(6%), 187(4%), 159(26%), 134(67%), 132(25%), 121(30%), 119(63%), 105(50%), 93(63%), 91(53%), 79(100%), 67(45%), 59(39%), 43(31%). ^g^ MS: 218(3%), 203(3%), 175(13%), 148(36%), 135(25%), 121(18%), 109(45%), 69(100%), 41(77%). ^h^ MS: 220(5%), 105(8%), 202(10%), 187(12%), 179(33%), 161(35%), 127(74%), 123(90%), 109(100%), 95(66%), 93(47%), 81(93%), 69(68%), 55(94%), 43(85%), 41(85%). ^i^ MS: 281(0.5%), 263(1%), 179(1%), 163(3%), 149(8%), 140(8%), 121(9%), 111(20%), 109(10%), 97(28%), 95(17%), 84(100%), 71(25%), 69(25%), 57(28%), 55(28%), 43(40%), 41(27%).

**Table 5:** Chemical compositions of *Callicarpa longifolia* leaf essential oils from Vietnam.

RI ^a^	RI ^b^	Compound	%	
			Da Nang	Nghia Dan
931	932	α-Pinene	0.4	0.1
1007	1008	δ-3-Carene	---	tr ^c^
1022	1024	*p*-Cymene	---	tr
1028	1024	Limonene	0.5	0.1
1033	1032	(*Z*)-β-Ocimene	---	tr
1043	1044	(*E*)-β-Ocimene	---	tr
1097	1095	Linalool	---	1.0
1101	1104	Hotrienol	---	tr
1103	1100	Nonanal	---	tr
1105	1110	Octen-3-yl acetate	---	tr
1110	1113	4,8-Dimethylnona-1,3,7-triene	---	tr
1116	1118	3-Octyl acetate	---	tr
1190	1190	Methyl salicylate	---	0.9
1192	1197	(2*E*)-Hexenyl butyrate	---	tr
1193	1186	α-Terpineol	---	0.1
1221	1227	Nerol	---	tr
1234	1226	(2*E*)-Hexenyl 2-methylbutyrate	---	tr
1247	1249	Geraniol	---	0.1
1290	1287	Dihydroedulan IIA	---	0.1
1295	1294	(*Z*)-Theaspirane	---	0.2
1311	1298	(*E*)-Theaspirane	---	0.2
1330	1334	Bicycloelemene	0.2	0.4
1333	1335	δ-Elemene	0.1	2.7
1349	1352	Tricyclosantalal A	---	0.2
1374	1374	α-Copaene	0.4	0.6
1375	1383	(*E*)-β-Damascenone	---	0.2
1387	1389	β-Elemene	0.5	0.4
1401	1408	(*Z*)-Caryophyllene	---	0.1
1403	1409	α-Gurjunene	---	0.2
1407	1415	β-Maaliene	0.7	---
1409	1411	*cis*-α-Bergamotene	---	0.1
1418	1417	(*E*)-Caryophyllene	11.8	28.0
1427	1434	γ-Elemene	0.6	1.3
1429	1432	*trans*-α-Bergamotene	---	0.5
1431	1438	α-Maaliene	---	0.1
1436	1439	Aromadendrene	0.2	0.5
1438	1442	6,9-Guaiadiene	---	0.5
1447	1445	Myltayl-4(12)-ene	0.7	---
1449	1457	Sesquisabinene	---	0.3
1452	1454	(*E*)-β-Farnesene	1.6	---
1454	1452	α-Humulene	1.9	1.6
1458	1458	*allo*-Aromadendrene	1.4	0.8
1472	1478	γ-Muurolene	---	0.2
1473	1475	γ-Gurjunene	---	0.6
1479	1484	Germacrene D	0.3	0.2
1483	1488	δ-Selinene	---	0.7
1484	1476	β-Chamigrene	4.0	---
1488	1489	β-Selinene	3.2	13.2
1489	1491	Eremophilene	4.3	---
1492	1500	Bicyclogermacrene	---	5.9
1494	1496	Valencene	1.4	---
1494	1500	α-Muurolene	---	0.2
1500	1502	*trans*-β-Guaiene	22.2	0.4
1510	1505	β-Bisabolene	1.2	0.3
1504	1507	Eremophila-1(10),8,11-triene	0.3	---
1511	1513	γ-Cadinene	0.2	0.2
1510	1508	6-*epi*-Shyobunone	0.3	---
1516	1522	δ-Cadinene	0.4	0.2
1520	1520	7-*epi*-α-Selinene	3.5	---
1535	1540	Selina-4(15),7(11)-diene	0.2	0.3
1538	1544	α-Calacorene	---	0.2
1557	1559	Germacrene B	1.3	2.1
1557	1561	(*E*)-Nerolidol	---	0.1
1570	1567	Palustrol	0.9	---
1576	1577	Spathulenol	1.1	5.3
1581	1582	Caryophyllene oxide	1.7	6.1
1584	1590	Globulol	0.2	0.2
1593	1592	Viridiflorol	0.3	0.2
1598	1596	*trans*-β-Elemenone	0.6	---
1605	1602	Ledol	2.4	---
1606	---	Unidentified ^d^	2.5	---
1610	1608	Humulene epoxide II	0.3	---
1616	---	Unidentified ^e^	0.2	2.7
1623	1624	Selina-6-en-4β-ol	---	0.4
1627	1629	*iso*-Spathulenol	0.2	4.2
1629	1642	Caryophylla-4(12),8(13)-dien-5α-ol	---	0.6
1632	1637	Dehydroxycalamendiol	0.7	---
1634	1644	Caryophylla-4(12),8(13)-dien-5β-ol	---	0.2
1652	1649	β-Eudesmol	---	0.9
1655	1652	α-Cadinol	0.7	---
1662	1658	Selin-11-en-4α-ol	8.0	7.4
1668	---	Unidentified ^f^	1.4	---
1670	---	Unidentified ^g^	1.2	---
1679	1685	Germacra-4(15),5,10(14)-trien-1α-ol	---	0.4
1685	1685	α-Bisabolol	0.8	---
1686	1690	(*Z*)-*trans*-α-Bergamotol	---	0.5
1693	1693	Germacrone	2.7	---
1704	1706	(*E*)-*trans*-α-Bergamotol	---	0.3
1711	1715	Pentadecanal	---	0.3
1723	1729	Isobicyclogermacrenal	0.7	---
1738	1734	1(10),11-Eremophiladien-9-one	6.7	---
1747	1744	Isocalamenediol	0.5	---
1765	1766	β-Costol	---	0.4
1768	1773	α-Costol	---	0.4
1777	1786	*trans*-Isovalencenol	0.2	---
1886	1891	(*E*)-Hexadecantrienal	---	0.2
2045	2046	Kaur-16-ene	0.3	---
2101	2109	(*E*)-Phytol	---	0.5
		Monoterpene hydrocarbons	0.9	0.1
		Oxygenated monoterpenoids	0.0	1.2
		Sesquiterpene hydrocarbons	63.0	62.9
		Oxygenated sesquiterpenoids	29.4	27.8
		Diterpenoids	0.3	0.5
		Others	0.0	2.3
		Total identified	93.5	94.8

^a^ RI = Retention Index determined with respect to a homologous series of n-alkanes on a ZB-5 column. ^b^ Retention indices from the databases. ^c^ tr = trace (<0.05%). ^d^ MS: 220(10%), 205(20%), 178(21%), 177 (47%), 153(19%), 140(30%), 135(20%), 121(17%), 107(47%), 97(100%), 93(44%), 81(73%), 79(65%), 69(57%), 67(31%), 55(56%), 41(53%). ^e^ MS: 222(3%), 207(38%), 204(42%), 189(40%), 161(33%), 147(25%), 137(27%), 135(55%), 133(25%), 121(28%), 109(42%), 107(35%), 105(26%), 95(47%), 93(41%), 81(94%), 71(54%), 67(41%), 55(41%), 43(100%), 41(39%). ^f^ MS: 220(16%), 205(96%), 202(20%), 187(35%), 177(35%), 163(40%), 159(100%), 151(50%), 149(40%), 145(57%), 131(52%), 121(59%), 119(88%), 109(55%), 107(81%), 105(85%), 93(98%), 91(90%), 79(70%), 67(69%), 55(73%), 41(73%). ^g^ MS: 220(3%), 205(64%), 189(33%), 177(21%), 162(29%), 147(100%), 138(40%), 133(66%), 119(45%), 109(44%), 107(50%), 105(64%), 93(73%), 91(79%), 79(71%), 67(54%), 55(60%), 41(57%).

**Table 6:** Chemical composition of *Callicarpa nudiflora* leaf essential oil from Son Tra Peninsula, Da Nang City, Vietnam.

RI ^a^	RI ^b^	Compound	%
920	921	Tricyclene	tr ^c^
923	924	α-Thujene	0.4
931	932	α-Pinene	8.1
945	945	α-Fenchene	tr
947	946	Camphene	0.5
951	953	Thuja-2,4(10)-diene	tr
970	969	Sabinene	0.6
977	974	β-Pinene	34.2
983	979	Octan-3-one	tr
989	988	Myrcene	0.2
988	988	Dehydro-1,8-cineole	tr
995	988	3-Octanol	tr
1023	1024	*p*-Cymene	2.3
1027	1024	Limonene	1.0
1029	1025	β-Phellandrene	0.1
1030	1026	1,8-Cineole	1.1
1033	1032	(*Z*)-β-Ocimene	0.1
1098	1099	α-Pinene oxide	0.4
1117	1114	*endo*-Fenchol	0.2
1123	1118	*cis*-*p*-Menth-2-en-1-ol	0.1
1125	1122	α-Campholenal	0.3
1137	1135	Nopinone	0.5
1139	1135	*trans*-Pinocarveol	2.0
1141	1136	*trans*-*p*-Menth-2-en-1-ol	tr
1144	1140	*trans*-Verbenol	0.1
1153	1145	Camphene hydrate	0.1
1156	1154	Sabina ketone	0.1
1159	1158	*trans*-Pinocamphone	tr
1160	1160	Pinocarvone	0.3
1170	1165	Borneol	0.2
1179	1174	Terpinen-4-ol	1.0
1185	1183	Cryptone	tr
1186	1179	*p*-Cymen-8-ol	0.2
1187	1182	*cis*-Pinocarveol	tr
1194	1195	Myrtenal	6.8
1217	1215	*trans*-Carveol	0.1
1273	1266	*trans*-Ascaridol glycol	0.1
1274	1269	Perilla aldehyde	0.1
1276	1277	Phellandral	0.1
1281	1287	Bornyl acetate	0.1
1296	1295	Thujyl acetate	0.2
1297	1294	Perilla alcohol	0.4
1304	---	Unidentified ^d^	1.0
1320	1324	Myrtenyl acetate	0.1
1373	1374	α-Copaene	0.3
1376	1383	(*E*)-β-Damascenone	tr
1386	1389	β-Elemene	tr
1417	1417	(*E*)-Caryophyllene	2.9
1436	1439	Aromadendrene	0.4
1452	1452	α-Humulene	0.2
1457	1458	*allo*-Aromadendrene	1.4
1484	1491	Eremophilene	0.2
1486	1489	β-Selinene	0.1
1488	1496	Viridiflorene	0.1
1510	1513	γ-Cadinene	0.1
1575	1577	Spathulenol	2.9
1581	1582	Caryophyllene oxide	20.1
1583	1590	Globulol	0.2
1607	1608	Humulene epoxide II	0.5
1631	1642	Caryophylla-4(12),8(13)-dien-5α-ol	0.4
1634	1644	Caryophylla-4(12),8(13)-dien-5β-ol	1.7
1653	1656	14-Hydroxy-9-*epi*-(*Z*)-caryophyllene	0.8
1668	1668	14-Hydroxy-9-*epi*-(*E*)-caryophyllene	0.5
1677	1678	9-Tetradecyn-1-ol	0.1
1989	1987	Manoyl oxide	0.3
2103	2106	(*E*)-Phytol	0.4
		Monoterpene hydrocarbons	47.5
		Oxygenated monoterpenoids	14.6
		Sesquiterpene hydrocarbons	5.7
		Oxygenated sesquiterpenoids	27.1
		Diterpenoids	0.7
		Others	0.1
		Total identified	95.8

^a^ RI = Retention Index determined with respect to a homologous series of n-alkanes on a ZB-5 column. ^b^ Retention indices from the databases. ^c^ tr = trace (<0.05%). ^d^ MS: 135(10%), 119(12%), 107(18%), 93(36%), 92(51%), 91(45%), 79(45%), 69(100%), 55(30%), 53(31%), 43(27%), 41(78%).

**Table 7:** Chemical composition of *Callicarpa petelotii* leaf essential oil from Tay Giang District, Quang Nam province, Vietnam.

RI ^a^	RI ^b^	Compound	%
921	924	α-Thujene	tr ^c^
927	932	2-Methyl-5-isopropenylfuran	tr
929	932	α-Pinene	0.4
945	946	Camphene	tr
972	969	Sabinene	0.1
973	974	β-Pinene	0.4
975	974	1-Octen-3-ol	tr
981	979	Octan-3-one	tr
984	988	Myrcene	0.1
985	984	2-Pentylfuran	tr
993	988	3-Octanol	tr
1003	1002	α-Phellandrene	0.5
1005	1008	δ-3-Carene	tr
1013	1014	α-Terpinene	tr
1020	1024	*p*-Cymene	0.5
1025	1024	Limonene	0.4
1026	1025	β-Phellandrene	1.5
1031	1032	(*Z*)-β-Ocimene	0.9
1041	1044	(*E*)-β-Ocimene	0.1
1053	1054	γ-Terpinene	tr
1081	1086	Terpinolene	tr
1095	1095	Linalool	0.4
1099	1104	Hotrienol	0.1
1101	1100	Nonanal	tr
1109	1113	4,8-Dimethylnona-1,3,7-triene	tr
1124	1128	*allo*-Ocimene	tr
1141	1139	(*E*)-Tagetone	tr
1183	1183	Cryptone	tr
1188	1190	Methyl salicylate	0.4
1191	1186	α-Terpineol	0.1
1246	1249	Geraniol	tr
1274	1277	Phellandral	tr
1279	1287	Bornyl acetate	tr
1283	1287	Dihydroedulan IA	tr
1288	1294	Dihydroedulan IIA	tr
1293	1299	(*Z*)-Theaspirane	tr
1309	1303	(*E*)-Theaspirane	tr
1329	1335	δ-Elemene	tr
1341	1345	α-Cubebene	tr
1363	1369	Cyclosativene	tr
1370	1374	α-Copaene	0.1
1373	1383	(*E*)-β-Damascenone	tr
1378	1387	β-Bourbonene	0.1
1382	1390	7-*epi*-Sesquithujene	tr
1383	1389	β-Elemene	0.4
1408	1407	Longifolene	tr
1413	1417	(*E*)-Caryophyllene	2.7
1424	1430	β-Copaene	0.1
1429	1437	α-Guaiene	tr
1443	1447	*iso*-Germacrene D	tr
1447	1454	(*E*)-β-Farnesene	tr
1452	1452	α-Humulene	53.8
1456	1456	Nootkatene	tr
1467	1476	Selina-4,11-diene	0.1
1469	1478	γ-Muurolene	tr
1475	1484	Germacrene D	0.5
1483	1489	β-Selinene	4.0
1491	1498	α-Selinene	12.8
1511	1518	δ-Cadinene	0.1
1571	1577	Spathulenol	0.1
1575	1582	Caryophyllene oxide	2.0
1587	1590	*cis*-β-Elemenone	0.3
1592	1592	Humulene epoxide I	1.1
1604	1608	Humulene epoxide II	8.1
1626	1642	Caryophylla-4(12),8(13)-dien-5α-ol	1.5
1631	1644	Caryophylla-4(12),8(13)-dien-5β-ol	0.5
1648	1649	β-Eudesmol	0.3
1652	1658	Selin-11-en-4α-ol	1.3
1664	1656	14-Hydroxy-9-*epi*-(*Z*)-caryophyllene	0.1
1675	1685	Germacra-4(15),5,10(14)-trien-1α-ol	0.7
1679	1668	*epi*-Zizanone	0.3
1708	1715	Pentadecanal	0.1
2012	2026	(*E*,*E*)-Geranyl linalool	0.3
		Monoterpene hydrocarbons	4.8
		Oxygenated monoterpenoids	0.5
		Sesquiterpene hydrocarbons	74.7
		Oxygenated sesquiterpenoids	16.3
		Diterpenoids	0.3
		Others	0.5
		Total identified	97.0

^a^ RI = Retention Index determined with respect to a homologous series of n-alkanes on a ZB-5 column. ^b^ Retention indices from the databases. ^c^ tr = trace (<0.05%).

**Table 8:** Chemical compositions of *Callicarpa rubella* leaf essential oils from Vietnam.

RI ^a^	RI ^b^	Compound	%		
			Nam Giai	Bach Ma	Tay Giang
923	924	α-Thujene	---	0.1	tr ^c^
930	932	α-Pinene	0.1	0.5	1.1
946	946	Camphene	---	---	tr
971	969	Sabinene	---	0.1	0.1
975	974	β-Pinene	0.3	2.4	1.7
977	974	1-Octen-3-ol	0.4	0.1	tr
982	979	3-Octanone	0.2	---	tr
986	988	Myrcene	---	0.1	0.2
987	984	2-Pentylfuran	---	---	tr
995	988	3-Octanol	0.4	0.1	tr
1005	1002	α-Phellandrene	---	0.3	3.0
1007	1008	δ-3-Carene	---	0.3	tr
1015	1014	α-Terpinene	---	---	tr
1022	1024	*p*-Cymene	tr	0.7	1.0
1027	1024	Limonene	0.1	0.4	0.8
1028	1025	β-Phellandrene	---	0.9	2.6
1030	1026	1,8-cineole	---	0.1	---
1033	1032	(*Z*)-β-Ocimene	---	---	0.1
1043	1044	(*E*)-β-Ocimene	---	---	tr
1055	1054	γ-Terpinene	---	---	tr
1067	1067	*cis*-Linalool oxide (furanoid)	0.2	---	---
1083	1086	Terpinolene	---	0.1	0.1
1084	1084	*trans*-Linalool oxide (furanoid)	0.2	---	---
1098	1095	Linalool	1.4	0.1	tr
1104	1100	Nonanal	---	0.1	tr
1123	1118	*cis-p*-Menth-2-en-1-ol	---	tr	---
1137	1134	Benzeneacetonitrile	0.1	---	---
1139	1135	*trans*-Pinocarveol	0.1	tr	---
1141	1136	*trans-p*-Menth-2-en-1-ol	---	tr	---
1161	1160	Pinocarvone	---	tr	---
1170	1165	Borneol	---	tr	---
1179	1174	Terpinen-4-ol	---	0.1	tr
1185	1183	Cryptone	---	0.2	---
1185	1184	(3*Z*)-Hexenyl butyrate	0.2	---	---
1189	1191	Hexyl butyrate	tr	---	---
1191	1190	Methyl salicylate	0.2	---	tr
1192	1193	(2*E*)-Hexenyl butyrate	0.1	---	---
1193	1195	Myrtenal	0.1	---	---
1194	1186	α-Terpineol	---	0.3	0.1
1201	1202	*cis*-Sabinol	---	0.1	---
1221	1222	2-Hydroxycineole	---	0.1	---
1285	1287	Dihydroedulan IA	0.1	---	tr
1290	1294	Dihydroedulan IIA	0.1	---	tr
1295	1299	(*Z*)-Theaspirane	---	---	0.1
1312	1303	(*E*)-Theaspirane	---	---	tr
1318	1318	3-Hydroxycineole	---	0.3	---
1328	1334	Bicycloelemene	---	0.2	0.3
1332	1335	δ-Elemene	---	0.2	0.2
1344	1345	α-Cubebene	0.1	0.4	17.4
1350	1356	Eugenol	---	---	0.1
1366	1373	α-Ylangene	0.1	---	tr
1372	1374	α-Copaene	0.4	0.1	4.6
1377	1383	(*E*)-β-Damascenone	---	---	tr
1380	1382	β-Bourbonene	3.2	0.1	4.1
1383	1385	α-Bourbonene	0.3	---	---
1384	1387	β-Cubebene	---	---	4.3
1386	1389	β-Elemene	0.5	1.3	0.5
1400	1408	(*Z*)-Caryophyllene	---	---	0.1
1403	1409	α-Gurjunene	---	---	0.1
1414	1419	β-Ylangene	0.3	---	---
1417	1417	(*E*)-Caryophyllene	0.3	7.1	18.0
1426	1430	β-Copaene	0.3	---	0.7
1426	1427	γ-Elemene	---	2.5	---
1430	1432	*trans*-α-Bergamotene	---	0.1	---
1431	1437	α-Guaiene	2.8	---	3.0
1435	1439	Aromadendrene	0.1	---	0.3
1441	1447	*iso*-Germacrene D	0.2	---	0.3
1445	1448	*cis*-Murrola-3,5-diene	---	---	0.6
1449	1454	(*E*)-β-Farnesene	---	0.5	0.1
1452	1452	α-Humulene	0.1	0.9	2.0
1456	1458	*allo*-Aromadendrene	---	---	0.2
1459	1465	*cis*-Muurola-4(14),5-diene	---	---	0.1
1465	1461	*cis*-Cadina-1(6),4-diene	---	---	0.2
1466	1473	Drima-7,9(11)-diene	0.1	---	---
1468	1475	*trans*-Cadina-1(6),4-diene	---	---	0.8
1471	1478	γ-Muurolene	0.5	---	0.5
1476	1475	γ-Gurjunene	0.2	---	0.4
1478	1479	*ar*-Curcumene	---	0.7	---
1478	1484	Germacrene D	---	---	4.2
1486	1489	β-Selinene	1.6	0.9	0.8
1488	1495	γ-Amorphene	---	---	1.2
1492	1500	Bicyclogermacrene	---	---	4.6
1493	1498	α-Selinene	---	0.7	---
1495	1500	α-Muurolene	---	0.1	0.9
1495	---	Unidentified ^d^	2.7	---	---
1498	1509	α-Bulnesene	1.7	---	1.8
1500	1505	(*E*,*E*)-α-Farnesene	---	---	0.1
1505	1505	β-Bisabolene	0.7	25.0	0.1
1510	1513	γ-Cadinene	---	0.1	0.3
1512	1514	Cubebol	---	0.5	1.0
1515	1518	δ-Cadinene	---	0.2	4.6
1519	1521	*trans*-Calamenene	---	0.2	0.4
1520	1528	Zonarene	---	---	0.2
1521	1521	β-Sesquiphellandrene	---	0.5	---
1529	1533	*trans*-Cadina-1,4-diene	---	---	0.4
1534	1540	Selina-4(15),7(11)-diene	---	0.5	---
1539	1545	Selina-3,7(11)-diene	---	0.3	---
1545	1548	α-Elemol	---	0.2	---
1556	1559	Germacrene B	---	4.6	0.1
1564	---	Unidentified ^e^	3.2	---	---
1568	---	Unidentified ^f^	7.2	---	---
1574	1577	Spathulenol	3.9	0.2	2.7
1579	1582	Caryophyllene oxide	25.1	3.0	2.7
1581	---	Unidentified ^g^	1.7	---	0.3
1590	1590	*cis*-β-Elemenone	---	0.4	---
1595	1596	*trans*-β-Elemenone	---	4.2	---
1607	1608	Humulene epoxide II	3.8	0.4	0.2
1625	1629	*iso*-Spathulenol	---	0.6	---
1625	1627	1-*epi*-Cubenol	---	---	0.8
1634	1644	Caryophylla-4(12),8(13)-dien-5β-ol	---	---	0.2
1640	1645	Cubenol	---	---	0.5
1641	1640	τ-Muurolol	---	---	0.1
1644	1644	α-Muurolol (= δ-Cadinol)	---	---	0.3
1653	1652	α-Cadinol	---	0.4	0.2
1655	1651	Pogostol	1.6	---	0.2
1661	---	Unidentified ^h^	---	2.2	---
1668	1668	14-Hydroxy-9-*epi*-(*E*)-caryophyllene	1.2	---	0.1
1677	---	Unidentified ^i^	1.1	---	---
1684	1685	α-Bisabolol	---	0.2	---
1691	1693	Germacrone	---	22.1	---
1698	1704	*cis*-Thujopsenol	8.8	---	---
1709	---	Unidentified ^j^	---	1.8	---
1715	---	Unidentified ^k^	2.1	---	---
1766	---	Unidentified ^l^	1.8	---	---
1768	---	Unidentified ^m^	---	1.0	---
1792	---	Unidentified ^n^	4.0	---	---
1802	---	Unidentified ^o^	2.5	---	---
1809	1806	Nootkatone	1.6	---	---
1815	---	Unidentified ^p^	---	1.8	---
1834	---	Unidentified ^q^	---	3.0	---
1849	---	Unidentified ^r^	1.6	---	---
1885	1884	Corymbolone	5.6	---	---
2049	2055	Abietatriene	---	1.3	0.1
		Monoterpene hydrocarbons	0.5	5.9	10.5
		Oxygenated monoterpenoids	2.0	1.2	0.1
		Sesquiterpene hydrocarbons	13.5	47.1	78.5
		Oxygenated sesquiterpenoids	45.9	32.3	9.0
		Diterpenoids	5.6	1.3	0.1
		Others	1.8	0.2	0.1
		Total identified	69.2	88.0	98.3

^a^ RI = Retention Index determined with respect to a homologous series of n-alkanes on a ZB-5 column. ^b^ Retention indices from the databases. ^c^ tr = trace (<0.05%). ^d^ MS: 202(24%), 189(12%), 187(14%), 159(28%), 147(66%), 145(53%), 134(30%), 133(31%), 131(35%), 121(37%), 119(59%), 107(68%), 105(99%), 93(92%), 91(79%), 81(100%), 80(69%), 79(74%), 77(42%), 67(35%), 55(40%), 41(55%). ^e^ MS: 220(3%), 205(13%), 187(18%), 162(34%), 147(27%), 145(30%), 135(35%), 121(67%), 107(85%), 95(74%), 93(77%), 81(81%), 79(67%), 69(68%), 67(82%), 55(87%)41(100%). ^f^ MS: 220(0.5%), 205(4%), 187(12%), 177(6%), 162(9%), 149(9%), 147(20%), 145(13%), 123(26%), 122(24%), 111(44%), 107(78%), 95(42%), 93(42%), 83(40%), 81(58%), 79(38%), 67(47%), 55(43%), 43(100%), 41(49%). ^g^ MS: 220(3%), 205(4%), 202(12%), 187(24%), 159(25%), 146(29%), 145(23%), 133(22%), 131(20%), 123(18%), 121(19%0, 119(18%), 109(18%), 107(27%), 105(28%), 95(29%), 93(35%), 91(30%), 81(35%), 79(36%), 69(27%), 55(32%), 43(100%), 41(33%). ^h^ MS: 218(4%), 203(5%), 175(14%), 136(68%), 135(68%), 121(25%), 107(100%), 91(29%), 79(22%), 67(50%), 53(18%), 41(28%). ^i^ MS: 218(7%), 203(8%), 175(12%), 161(17%), 160(18%), 147(22%), 145(15%), 135(28%), 134(29%), 121(32%), 119(33%), 109(42%), 107(35%), 105(33%), 95(59%), 93(41%), 81(34%), 79(30%), 69(30%), 67(37%),55(29%), 53(19%), 43(100%), 41(43%). ^j^ MS: 220(35%), 205(17%), 202(27%), 187(27%), 162(88%), 159(32%), 149(55%), 147(57%), 145(30%), 131(30%), 121(71%), 119(79%), 107(66%), 105(68%), 97(43%), 93(57%), 91(67%), 43(100%), 41(63%). ^k^ MS: 218(2%), 200(3%), 185(5%), 160(13%), 145(9%), 121(25%), 120(27%), 98(22%), 97(23%), 83(100%), 67(18%), 55(34%), 43(96%), 41(25%). ^l^ MS: 220(1%), 205(4%), 179(10%), 161(4%), 147(10%), 137(10%), 133(14%), 121(17%), 119(20%), 108(32%), 95(35%), 93(53%), 91(42%), 81(34%), 79(61%), 69(32%), 67(38%), 55(45%), 43(100%), 41(58%). ^m^ MS: 220(62%), 202(11%), 187(15%), 177(100%), 159(74%), 135(49%), 123(75%), 107(76%), 93(64%), 91(52%), 81(61%), 67(54%), 55(57%), 43(62%), 41(68%). ^n^ MS: 234(8%), 219(30%), 216(8%), 201(8%), 191(12%), 177(15%), 176(13%), 163(14%), 159(16%), 152(21%), 137(16%), 133(16%), 111(27%), 105(20%), 91(25%), 79(19%), 77(17%), 67(17%), 55(17%), 43(100%), 41(26%). ^o^ MS: 234(1%), 216(16%), 188(8%), 177(9%), 173(8%), 163(10%), 161(14%), 159(13%), 133(22%), 111(26%), 105(24%), 95(47%), 91(23%), 81(23%), 79(24%), 77(20%), 67(22%), 55(18%), 43(100%), 41(28%). ^p^ MS: 234(2%), 219(4%), 201(5%), 191(5%), 177(7%), 167(12%), 149(34%), 135(51%), 121(50%), 107(100%), 93(35%), 91(37%), 79(34%), 68(36%), 67(58%), 55(34%), 43(53%), 41(60%). ^q^ MS: 167(40%), 121(32%), 68(100%), 67(58%), 43(33%), 41(39%). ^r^ MS: 236(5%), 221(8%), 218(8%), 193(15%), 180(26%), 167(30%), 149(22%), 147(18%), 136(65%), 123(74%), 110(97%), 97(84%), 69(83%), 55(80%), 43(80%), 41(100%).

**Table 9:** Chemical composition of *Callicarpa rubella* stem bark essential oil from Bach Ma National Park, Vietnam.

RI ^a^	RI ^b^	Compound	%
933	932	α-Pinene	0.4
949	946	Camphene	0.1
972	969	Sabinene	0.1
978	974	β-Pinene	2.7
989	988	Myrcene	0.1
1007	1002	α-Phellandrene	0.3
1009	1008	δ-3-Carene	1.5
1025	1024	*p*-Cymene	0.6
1029	1024	Limonene	0.4
1031	1025	β-Phellandrene	0.7
1085	1086	Terpinolene	0.1
1100	1095	Linalool	0.1
1196	1186	α-Terpineol	0.1
1333	1334	Bicycloelemene	0.1
1347	1345	α-Cubebene	0.7
1376	1374	α-Copaene	0.2
1382	1389	β-Elemene	0.1
1388	1387	β-Cubebene	0.3
1389	1389	β-Elemene	2.0
1420	1417	(*E*)-Caryophyllene	7.3
1429	1427	γ-Elemene	4.8
1452	1457	Sesquisabinene	0.3
1456	1452	α-Humulene	1.0
1460	1458	*allo*-Aromadendrene	0.1
1472	1475	*trans*-Cadina-1(6),4-diene	0.2
1481	1479	*ar*-Curcumene	2.2
1489	1489	β-Selinene	0.6
1492	1493	*trans*-Muurola-4(14),5-diene	0.2
1496	1498	α-Selinene	1.0
1498	1500	α-Muurolene	0.3
1505	1501	Aciphyllene	0.3
1508	1505	β-Bisabolene	17.9
1513	1513	γ-Cadinene	0.3
1515	1514	Cubebol	0.3
1518	1518	δ-Cadinene	0.5
1521	1521	*trans*-Calamenene	0.2
1524	1521	β-Sesquiphellandrene	0.6
1537	1528	Zonarene	0.6
1542	1545	Selina-3,7(11)-diene	0.5
1548	1548	Elemol	0.3
1559	1559	Germacrene B	8.4
1577	1577	Spathulenol	0.2
1582	1582	Caryophyllene oxide	1.9
1593	1590	*cis*-β-Elemenone	0.4
1594	1592	Viridiflorol	0.4
1598	1596	*trans*-β-Elemenone	3.8
1607	1608	β-Atlantol	0.2
1610	1608	Humulene epoxide II	0.1
1628	1629	*iso*-Spathulenol	0.5
1632	1630	γ-Eudesmol	0.1
1643	1645	Cubenol	0.3
1647	1644	α-Muurolol (=δ-Cadinol)	0.2
1655	1652	α-Cadinol	0.7
1658	1658	Selin-11-en-4α-ol	0.1
1664	---	Unidentified ^c^	1.6
1687	1685	α-Bisabolol	0.4
1694	1693	Germacrone	23.9
2015	2009	13-*epi*-Manool oxide	0.2
2053	2055	Abietatriene	1.1
		Monoterpene hydrocarbons	7.1
		Oxygenated monoterpenoids	0.9
		Sesquiterpene hydrocarbons	50.7
		Oxygenated sesquiterpenoids	36.1
		Diterpenoids	1.3
		Others	0.0
		Total identified	93.0

^a^ RI = Retention Index determined with respect to a homologous series of n-alkanes on a ZB-5 column. ^b^ Retention indices from the databases. ^c^ MS: 218(5%), 203(7%), 185(5%), 175(18%), 161(4%), 147(10%), 136(77%), 135(78%), 121(33%), 107(100%), 91(29%), 79(21%), 67(44%), 55(11%), 53(17%), 41(24%).

**Table 10:** Chemical composition of *Callicarpa sinuata* leaf essential oil from Son Tra Peninsula, Da Nang City, Vietnam.

RI ^a^	RI ^b^	Compound	%
930	932	α-Pinene	0.1
969	969	Sabinene	0.1
975	974	β-Pinene	tr ^c^
976	974	1-Octen-3-ol	tr
1006	1008	δ-3-Carene	tr
1022	1024	*p*-Cymene	0.1
1026	1024	Limonene	0.1
1030	1026	1,8-Cineole	tr
1103	1100	Nonanal	0.1
1295	1299	(*Z*)-Theaspirane	0.1
1311	1303	(*E*)-Theaspirane	0.1
1328	1334	Bicycloelemene	0.1
1331	1335	δ-Elemene	0.3
1343	1345	α-Cubebene	2.0
1365	1373	α-Ylangene	0.1
1372	1374	α-Copaene	12.6
1378	1383	*cis*-β-Elemene	0.2
1380	1382	β-Bourbonene	0.3
1384	1387	β-Cubebene	1.8
1385	1389	*trans*-β-Elemene	3.1
1416	1417	(*E*)-Caryophyllene	3.8
1426	1430	β-Copaene	0.4
1431	1437	α-Guaiene	0.2
1435	1439	Aromadendrene	0.5
1448	1455	Valerena-4,7(11)-diene	0.4
1453	1452	α-Humulene	24.8
1456	1458	*allo*-Aromadendrene	0.4
1466	1473	4,5-di-*epi*-Aristolochene	0.4
1469	1476	Selina-4,11-diene	0.2
1471	1478	γ-Muurolene	1.3
1475	1483	α-Amorphene	0.2
1477	1484	Germacrene D	2.6
1484	1491	Eremophilene	0.3
1485	1489	β-Selinene	1.8
1488	1493	*trans*-Muurola-4(14),5-diene	0.3
1492	1500	Bicyclogermacrene	4.0
1494	1500	α-Muurolene	0.5
1509	1513	γ-Cadinene	0.6
1511	1514	Cubebol	0.4
1514	1518	δ-Cadinene	2.3
1517	1521	*trans*-Calamenene	0.4
1538	1544	α-Calcorene	0.9
1556	1561	(*E*)-Nerolidol	0.3
1558	1564	β-Calacorene	0.4
1573	1577	Spathulenol	5.9
1578	1582	Caryophyllene oxide	1.9
1582	1590	Globulol	0.4
1590	1592	Viridiflorol	0.5
1592	1600	Guaiol	0.2
1595	1592	Humulene epoxide I	0.6
1606	1608	Humulene epoxide II	6.7
1624	1627	1-*epi*-Cubenol	0.7
1628	1629	*iso*-Spathulenol	2.4
1639	1638	τ-Cadinol	0.3
1640	1640	τ-Muurolol	0.3
1651	1652	α-Eudesmol	2.3
1655	1658	Selin-11-en-4α-ol	0.7
1659	1668	*ar*-Turmerone	0.2
1661	1662	9-Methoxycalamenene	0.5
1669	1675	Cadalene	0.2
1734	1740	Mint sulfide	1.2
1836	1841	Phytone	0.2
2048	2055	Abietatriene	0.1
2101	2106	(*E*)-Phytol	3.4
2132	2138	Palmitaldehyde, diallyl acetal	0.6
		Monoterpene hydrocarbons	0.3
		Oxygenated monoterpenoids	tr
		Sesquiterpene hydrocarbons	67.5
		Oxygenated sesquiterpenoids	24.1
		Diterpenoids	3.8
		Others	2.1
		Total identified	97.8

^a^ RI = Retention Index determined with respect to a homologous series of *n*-alkanes on a ZB-5 column. ^b^ Retention indices from the databases. ^c^ tr = trace (<0.05%).

**Table 11:** Twenty-four-hour mosquito larvicidal activity (µg/mL) of *Callicarpa* leaf essential oils from central Vietnam.

*Callicarpa* Species	LC_50_ (95% Confidence Limits)	LC_90_ (95% Confidence Limits)	χ^2^	*p*
	*Aedes aegypti*			
*C. bodinieri*	53.99 (50.29–58.32)	76.61 (69.03–90.35)	4.90	0.086
*C. candicans* (Nghia Dan)	5.337 (4.769–5.961)	12.05 (10.38–14.57)	10.10	0.018
*C. candicans* (Dai Loc)	2.695 (2.342–3.051)	6.633 (5.685–8.107)	70.57	0.000
*C. formosana*	31.85 (29.39–34.55)	48.94 (44.06–56.50)	3.74	0.154
*C. longifolia* (Nghia Dan)	37.44 (34.16–41.05)	66.33 (58.53–78.54)	3.86	0.145
*C. nudiflora*	37.51 (33.76–41.79)	79.16 (67.91–97.04)	9.47	0.009
*C. petelotii*	19.14 (17.13–21.22)	37.87 (32.85–46.26)	8.37	0.015
*C. rubella* (Nam Giai)	24.15 (21.33–27.13)	57.15 (48.61–71.14)	3.22	0.200
*C. rubella* (Tay Giang)	26.00 (24.19–28.06)	39.42 (34.57–47.24)	2.43	0.297
*C. sinuata*	28.69 (25.87–31.82)	58.15 (50.38–70.31)	7.43	0.024
	*Culex quinquefasciatus*			
*C. candicans* (Nghia Dan)	2.041 (1.683–2.426)	10.43 (8.14–14.46)	5.36	0.252
*C. candicans* (Dai Loc)	1.204 (0.903–1.510	7.841 (6.035–11.146)	2.01	0.734
*C. nudiflora*	108.9 (101.2–117.1)	75.76 (66.11–85.11)	2.34	0.126

**Table 12:** Forty-eight-hour mosquito larvicidal activity (µg/mL) of *Callicarpa* leaf essential oils from central Vietnam.

*Callicarpa* Species	LC_50_ (95% Confidence Limits)	LC_90_ (95% Confidence Limits)	χ^2^	*p*
	*Aedes aegypti*			
*C. bodinieri*	52.00 (48.39–56.11)	74.18 (66.88–87.49)	2.43	0.297
*C. candicans* (Nghia Dan)	3.824 (3.426–4.256)	8.165 (7.077–9.813)	17.80	0.000
*C. candicans* (Dai Loc)	2.145 (1.998–2.301)	2.891 (2.667–3.211)	8.14	0.012
*C. formosana*	29.04 (26.89–31.49)	43.37 (39.04–50.31)	9.12	0.010
*C. longifolia* (Nghia Dan)	35.64 (32.40–39.22)	66.15 (58.04–78.86)	6.87	0.032
*C. nudiflora*	27.34 (23.84–31.16)	77.02 (63.21–101.27)	17.10	0.000
*C. petelotii*	18.49 (16.52–20.50)	36.52 (31.69–44.62)	6.65	0.036
*C. rubella* (Nam Giai)	17.93 (14.85–20.87)	54.72 (45.00–72.37)	3.61	0.165
*C. rubella* (Tay Giang)	21.73 (19.75–23.90)	37.09 (33.44–43.74)	8.48	0.014
*C. sinuata*	25.86 (23.20–28.77)	54.55 (47.03–66.51)	5.20	0.074
	*Culex quinquefasciatus*			
*C. candicans* (Nghia Dan)	1.670 (1.425–1.929)	5.726 (4.688–7.448)	16.78	0.002
*C. candicans* (Dai Loc)	0.945 (0.742–1.137)	3.537 (2.881–4.691)	9.68	0.046
*C. nudiflora*	178.5 (148.3–240.1)	170.6 (153.7–198.8)	15.72	0.000

**Table 13:** Collection details and essential oil yields of *Callicarpa* species from central Vietnam.

*Callicarpa* Species	Vietnamese Name	Collection Site	Growth Period	Voucher Number	Part	% Yield
*Callicarpa bodinieri* Lév.	Tử châu bodinier	Ngoc Linh Nature Reserve, Quang Nam Province (15°50′16.0″ N, 107°22′54.7″ E, elev. 1341 m)	Flowers and young fruits	DND-62	Leaf	0.1
*Callicarpa candicans* (Burm.f.) Hochr.	Tử châu chồi trắng, Nàng nàng	Nghia Dan District, Nghe An province (19°22′24.4″ N, 105°25′15.3″ E, elev. 75 m)	Flowers, young fruits and ripe fruits	DND-17	Leaf	0.15
		Dai Loc district, Quang Nam province 15°53′16″ N, 107°59′38″ E, elev. 514 m)	Flowers, young fruits and ripe fruits	DND-80	Leaf	0.18
		Hoa Vang district, Da Nang city (16°01′0.6″ N, 108°4′25.6″ E, elev. 28 m)	Flowers, young fruits and ripe fruits	NHH-57	Leaf Bark	0.17 0.04
*Callicarpa formosana* Rolfe	Tử châu đài loan	Ngoc Linh Nature Reserve, Quang Nam Province (15°50′16.0″ N, 107°22′54.7″ E, elev. 1341 m)	Flowers and young fruits	DND-72	Leaf	0.11
*Callicarpa longifolia* Lam.	Tử châu lá dài, Tu hú lá dài	Nghia Dan District, Nghe An province (19°20′6.2″ N, 105°25′58.1″ E, elev. 51 m)	Flowers, young fruits and ripe fruits	DND-31	Leaf	0.13
*Callicarpa nudiflora* Hook. & Arn.	Tử châu hoa trần	Son Tra Peninsula, Da Nang City (16°07′18″ N, 108°18′07″ E, elev. 118 m)	Flowers, young fruits and ripe fruits	DND-33	Leaf	0.14
*Callicarpa petelotii* Dop	Tử châu petelot	Tay Giang District, Quang Nam province (15°50′16.0″ N, 107°22′54.7″ E, elev. 1341 m)	Flowers, young fruits and ripe fruits	DND-98	Leaf	0.22
*Callicarpa rubella* Lindl.	Tử châu đỏ, Tu hú hồng	Nậm Giải Commune, Quế Phong district, Pu Hoat Nature Reserve, Nghe An province (19°41′40″ N, 104°49′29″ E, elev. 671 m)	Flowers, young fruits and ripe fruits	DND-709	Leaf	0.15
		Tay Giang District, Quang Nam province (15°50′16.0″ N, 107°22′54.7″ E, elev. 1341 m)	Flowers, young fruits	DND-99	Leaf	0.12
		Bach Ma National Park, Phu Loc District, Thua Thien Hue province (16°11′59″ N, 107°51′25″ E, elev. 1376 m)	ripe fruits	DND-27	Leaf Bark	0.11 0.06
*Callicarpa sinuata* Budantzev & Phuong	Tử châu răng sâu	Son Tra Peninsula, Da Nang City (16°06′00″ N, 108°18′24″ E, elev. 124 m)	Flowers, young fruits	NHH-84	Leaf	0.14
